# Lineage Plasticity and Histologic Transformation in EGFR-TKI Resistant Lung Cancer

**DOI:** 10.3390/ijms27010445

**Published:** 2025-12-31

**Authors:** Li Yieng Eunice Lau, Anders Jacobsen Skanderup, Aaron C. Tan

**Affiliations:** 1 Genome Institute of Singapore (GIS), Agency for Science, Technology and Research (A*STAR), 60 Biopolis Street, Genome, Singapore 138672, Singapore; eunice_lau@a-star.edu.sg (L.Y.E.L.); skanderupamj@a-star.edu.sg (A.J.S.); 2 Yong Loo Lin School of Medicine, National University of Singapore, 10 Medical Drive, Singapore 117597, Singapore; 3 Division of Medical Oncology, National Cancer Centre Singapore, Singapore 168583, Singapore; 4 Duke-NUS Medical School, National University of Singapore, Singapore 169857, Singapore

**Keywords:** lineage plasticity, transcriptional reprogramming, epigenetic reprogramming, lung cancer, lung adenocarcinoma, EGFR-TKI, histologic transformation, acquired resistance, targeted therapy

## Abstract

Lineage plasticity, the ability of cancer cells to alter their differentiated state through transcriptional and epigenetic reprogramming, has emerged as a key mechanism of therapeutic resistance across cancers. This adaptive process can manifest in multiple ways, including epithelial–mesenchymal transition, acquisition of stem-like features, and histological transformation, the most striking and clinically apparent example. In *EGFR*-mutant lung adenocarcinoma (LUAD), lineage plasticity is increasingly recognized as a prevalent mechanism of acquired resistance to tyrosine kinase inhibitors (TKIs). Among its visible manifestations, histologic transformation into small-cell lung cancer (SCLC) is the most frequent, while squamous transformation and other phenotypic shifts also occur. Transformed tumors typically retain the initiating *EGFR* mutation but lose EGFR dependence, acquire neuroendocrine features, and display aggressive clinical behavior with poor clinical outcomes compared with both de novo SCLC and non-transformed LUAD. Recent studies show that plasticity arises through combined genomic, transcriptomic, and epigenetic reprogramming, often foreshadowed by molecular alterations before overt histological change. Spatial and single-cell profiling reveal heterogeneous trajectories and intermediate states, while functional models and multi-omics approaches have begun to identify therapeutic vulnerabilities distinct from both de novo *EGFR*-mutated SCLC and classical *EGFR*-mutated LUAD. Thus, lineage plasticity, whether manifested as histologic transformation or through more subtle epigenetic reprogramming, represents a formidable resistance mechanism in NSCLC. Defining its molecular basis and temporal dynamics will be essential for early detection, prognostication, and the development of tailored therapies.

## 1. Introduction

Non-small cell lung cancer (NSCLC) accounts for the majority of lung cancer cases, with lung adenocarcinoma (LUAD) being the most common subtype. A substantial proportion of LUADs are driven by activating mutations in the epidermal growth factor receptor (*EGFR*) tyrosine kinase domain, most notably exon 19 deletions and the L858R point mutation, which together comprise ~90% of *EGFR* mutations seen in clinical practice. These alterations confer marked sensitivity to EGFR tyrosine kinase inhibitors (TKIs), producing initial response rates of up to 80% [1,2,3,4,5,6,7].

Despite these remarkable responses, acquired resistance to EGFR-TKIs is inevitable, with a median progression-free survival (PFS) of approximately 18 months [2,7,8]. Resistance occurs across all TKI generations, first (erlotinib, gefitinib), second (afatinib, dacomitinib), and third (osimertinib), and is broadly classified as primary or acquired [9].

Primary resistance refers to the lack of objective response to EGFR-TKI or progressive disease as the best response, where patients fail to achieve any meaningful response and exhibit rapid disease progression within the first 6 months of therapy [9,10,11]. Acquired resistance, in contrast, denotes disease progression after an initial benefit, typically following ≥6 months of treatment, as defined by the Jackman criteria [12].

The T790M gatekeeper mutation remains the predominant acquired resistance mechanism, detected in ~60% of patients progressing on first- or second-generation EGFR-TKIs [13,14,15]. Third-generation inhibitors such as osimertinib were developed to overcome T790M-mediated resistance while retaining potent activity against sensitizing *EGFR* mutations. Following its superior PFS in the FLAURA trial (NCT02296125), osimertinib became the standard first-line therapy for *EGFR*-mutated advanced NSCLC [7,16].

Beyond monotherapy, third-generation EGFR-TKIs have been evaluated in combination regimens aimed at targeting complementary resistance pathways and improving response durability. In the FLAURA2 trial (NCT04035486), first-line osimertinib plus platinum–pemetrexed significantly improved PFS and overall survival (OS) compared with osimertinib alone [17], while the MARIPOSA trial (NCT04487080) demonstrated improved PFS with lazertinib (another third-gen TKI) plus amivantamab (EGFR/MET antibody) in untreated *EGFR*-mutant advanced NSCLC [18]. These rational combinations may modulate or delay resistance mechanisms but have yet to fully prevent therapeutic escape.

Aside from on-target mechanisms, such as *EGFR* T790M and C797S, acquired resistance can also arise through off-target or bypass signaling pathways (*MET* or *HER2* amplification) and lineage plasticity-driven processes such as epithelial–mesenchymal transition (EMT) and histologic transformation [19].

Lineage plasticity is the capacity of tumor cells to alter their differentiated state through transcriptional and epigenetic reprogramming. It encompasses a spectrum of adaptive behaviors, ranging from subtle transcriptional state shifts to overt histological transitions. Comprehensive reviews by Quintanal-Villalonga et al. (2020) and Thankamony et al. (2021) have synthesized these diverse manifestations, emphasizing the interplay of genetic drivers, epigenetic remodeling, and microenvironmental cues in enabling plastic adaptation across cancer types [20,21]. Within this broader framework, histologic transformation of *EGFR*-mutant NSCLC represents one of the clinically consequential forms of lineage plasticity in lung cancer.

Histologic transformation refers to the transition of LUAD into a different histologic subtype. This concept was first established in castration-resistant prostate cancer, where up to 20% of adenocarcinomas lose androgen receptor dependence and transdifferentiate into a neuroendocrine phenotype under the selective pressure of androgen deprivation therapy [22]. Analogous events occur in *EGFR*-mutant LUAD, where prolonged TKI treatment can lead to transformation into small-cell lung cancer (SCLC) or, less commonly, squamous cell carcinoma (SCC) [15,23]. Notably, *EGFR*-mutant NSCLCs that undergo SCLC transformation have poorer outcomes compared to their non-transformed counterparts, highlighting the aggressive clinical course associated with this adaptive phenotype [24]. Similar lineage plasticity-driven transitions have been described in ovarian and colorectal cancer, underscoring their role as a general adaptive strategy that enables tumors to evade therapy [25,26,27].

The clinical implications of transformation are profound. Transformed tumors typically display aggressive biological behavior, lose sensitivity to therapies effective against the original histology, and are associated with poor prognosis. Yet the true incidence remains underestimated, owing to practical and biological barriers. Re-biopsy at progression is often limited by patient condition, anatomical accessibility, or procedural risk, while small biopsies may miss tumor heterogeneity within or across lesions, including spatially distinct transformed clones [28]. Even when samples are available, the reliance on FFPE tissue provides only a static snapshot, which may miss earlier molecular reprogramming events that preceded overt morphological change [29,30].

Consequently, our understanding of histologic transformation and its molecular drivers is incomplete. Current clinical workflows largely prioritize known actionable mutations, potentially overlooking transcriptomic or epigenetic changes that underpin lineage plasticity.

In this review, we focus on histologic transformation in *EGFR*-mutant LUAD, emphasizing its basis in lineage plasticity. While our primary focus is on morphologically evident transformation, we also discuss emerging evidence that subtle molecular and epigenetic reprogramming may precede or occur independently of overt histologic change. We outline its clinical features and incidence, examine the molecular and cellular mechanisms that enable adenocarcinoma cells to adopt small-cell or squamous phenotypes, and discuss challenges in detection, including sampling bias and the limitations of current biopsy approaches. Finally, we consider the clinical implications of transformation, highlighting its impact on treatment resistance, prognosis, and emerging strategies for therapeutic intervention.

Taken together, histologic transformation represents a clinically significant yet underrecognized mechanism of resistance in *EGFR*-mutant LUAD. While most cases transform into SCLC, occasional transitions into squamous histology highlight the diverse manifestations of lineage plasticity. The clinical course of these patients is often aggressive, with limited therapeutic options and poor outcomes. A deeper understanding of the biological underpinnings and clinical features of transformation is therefore essential to improve detection, refine treatment strategies, and ultimately guide the development of novel interventions.

## 2. Historical Perspective and Prevalence

The importance of transformation becomes clearer when viewed through its historical recognition and reported frequency in *EGFR*-mutant LUAD.

Among the reported cases, small-cell lung cancer (SCLC) is by far the most frequent form of histological transformation [31]. In contrast, transformation into squamous cell carcinoma (SCC) has also been described, though most accounts are single case reports, making its true prevalence difficult to establish [32,33,34].

The first case of SCLC transformation was described in 2006 by Zakowski and colleagues in a 45-year-old, non-smoking woman [30]. Initially diagnosed with LUAD via transbronchial biopsy and bronchial wash examination, she achieved a partial response to erlotinib lasting 18 months before developing a new brain lesion. A subsequent lung biopsy revealed synaptophysin-positive SCLC harboring the same *EGFR* exon 19 deletion (L747-P753insQ) as the original tumor. Due to limited tissue availability, it was unclear whether SCLC-associated drivers were present prior to therapy. Importantly, despite retention of the *EGFR* mutation, the tumor was resistant to both EGFR-TKIs and standard SCLC chemotherapy, underscoring the loss of EGFR dependence and the aggressive biology of transformed SCLC compared with de novo disease [30]. This landmark observation set the stage for subsequent reports and ultimately for systematic studies that established SCLC transformation as a distinct, reproducible mechanism of acquired resistance in *EGFR*-mutant LUAD.

Following this report, additional cases of SCLC transformation in *EGFR*-mutant NSCLC were described, although many remain isolated case reports [35,36]. More systematic studies have since defined its prevalence, which ranges from ~2 to 14% of *EGFR*-mutant LUAD cases depending on the cohort and methodology [14,15,31,37]. In a prospective series by Sequist et al., 14% of patients (5 out of 37) demonstrated SCLC transformation while retaining their original *EGFR* mutation (L858R or exon 19 deletions) after progression on erlotinib [14]. In contrast, Yu et al. reported a lower rate of 3% (4 out of 155 patients) [15], while a larger study by Fujimoto et al. observed transformation in only 1.8% (48 out of 2624 cases), all confirmed by re-biopsy after resistance to EGFR-TKI therapy [37]. Chua et al. reported one case of squamous transformation (1.7%, 1 out of 59 patients) in a post-EGFR-TKI progressive sample that was adenocarcinoma at baseline [38].

These discrepancies likely reflect multiple sources of variability, including differences in cohort size, sampling depth, and the timing of the biopsy along the continuum of histologic transformation. Because lineage switching unfolds progressively, biopsies obtained at clinical progression may capture tumors at different stages of this process, ranging from molecularly reprogrammed but morphologically adenocarcinoma-like states to overt neuroendocrine transformation, leading to under-detection when histologic criteria alone are applied. Variability is further amplified by differences in analytic definitions of transformation, including reliance on histology versus transcriptional or immunohistochemical neuroendocrine signatures.

Together, these studies establish SCLC transformation as a reproducible, clinically significant mechanism of acquired resistance in *EGFR*-mutant LUAD, albeit with variable reported prevalence. Differences likely stem from cohort size, biopsy practices, and detection sensitivity, which will be further addressed in subsequent sections and are summarized in Table 1.

The recognition of SCLC transformation as a recurrent resistance phenotype has prompted investigation into its biological basis, particularly whether *EGFR*-mutant adenocarcinoma and SCLC derive from a shared progenitor population capable of lineage plasticity under therapeutic pressure.

## 3. Cell of Origin and Lineage Plasticity

The distinct histologic phenotypes of LUAD and SCLC have traditionally been attributed to differences in their cells of origin. LUAD is thought to arise predominantly from alveolar type II (AT2) cells, whereas SCLC has been linked to basal or neuroendocrine progenitor cells within the distal airway epithelium [47]. Yet lineage-tracing studies demonstrate that these boundaries are flexible. Under specific genetic contexts, particularly concurrent *TP53* and *RB1* inactivation, AT2 cells can acquire neuroendocrine features, giving rise to SCLC-like tumors [48].

Active *EGFR* signaling appears integral to maintaining AT2 identity; its inhibition, such as during EGFR-TKI therapy, can drive loss of epithelial phenotype [49,50]. When additional events such as TP53/RB1 inactivation occur, neuroendocrine trans-differentiation is therefore promoted. This mechanism explains both therapy-induced and de novo transformation in *EGFR*-mutant tumors. In de novo *EGFR*-mutant SCLC, it remains debated whether tumors originate directly from neuroendocrine progenitors or represent early transformation of AT2-derived precursors before diagnosis. Genomic evidence showing shared *EGFR* mutations with complete *TP53*/*RB1* loss supports the latter model, implying transformation independent of therapeutic pressure [40].

Consistent with this, Lee et al. reported that *EGFR*-mutant LUAD and transformed SCLC share a common clonal origin, with *TP53*/*RB1* inactivation preceding divergence [40]. These findings indicate that lineage plasticity is encoded early in tumor evolution, and that the interplay between AT2-cell origin and tumor-suppressor loss dictates whether *EGFR*-mutant LUAD retains adenocarcinoma identity or shifts toward an SCLC phenotype.

Collectively, these data establish lineage plasticity as a fundamental property of *EGFR*-mutant lung cancers, predisposing AT2-derived tumors to adopt neuroendocrine features under defined genetic or therapeutic pressures. This biological continuum has direct pathological implications, as distinguishing true transformation from pre-existing neuroendocrine components remains a central diagnostic challenge.

## 4. Clinical and Pathological Characteristics

Accurately confirming histologic transformation remains challenging. Although baseline diagnostic tissue is routinely available, the amount is often insufficient for comprehensive molecular profiling, limiting direct comparison with post-progression specimens. This constraint hampers efforts to determine whether transformation represents a true acquired resistance mechanism or the clonal expansion of pre-existing minor subclones under the selective pressure of EGFR-targeted therapy.

By contrast, studies with paired pre- and post-progression samples provide the most compelling evidence of true acquired transformation. In seminal work by Sequist et al. (2011), later corroborated by Yu et al. (2013) and Quintanal-Villalonga et al. (2021), transformed tumors retained their original *EGFR* mutations yet exhibited de novo expression of neuroendocrine markers, including synaptophysin, chromogranin A, and CD56, which were absent in the corresponding pre-treatment tissue [14,15,28]. Collectively, these findings support the interpretation that neuroendocrine differentiation emerges under therapeutic pressure, although such lineages may exist at low frequency prior to treatment and subsequently undergo clonal expansion following therapy.

**Figure 1:**
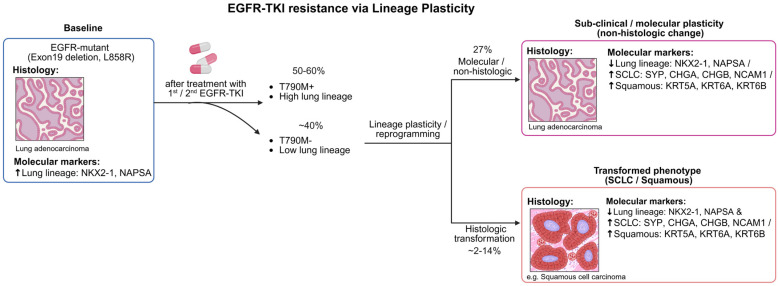
Divergent routes of resistance in *EGFR*-mutant lung adenocarcinoma. Following EGFR-TKI therapy, tumors may undergo lineage reprogramming that manifests either as molecular plasticity without overt histologic change or as complete histologic transformation to small-cell or squamous phenotypes. Both processes involve loss of lung-lineage markers and activation of alternative lineage programs, contributing to therapeutic resistance to EGFR-TKI. Created in BioRender. Skanderup, A. (2025) https://BioRender.com/fmfqlv7 (accessed on 15 November 2025).

Histologically, *EGFR*-mutant adenocarcinomas initially characterized by glandular differentiation and positive expression of markers such as TTF-1 and Napsin A undergo a pronounced shift upon transformation. Transformed tumors adopt a high-grade neuroendocrine phenotype, with a nest of small cells, scant cytoplasm, and a high nuclear-to-cytoplasmic ratio, consistent with classic SCLC morphology [14,20]. Immunohistochemically, they typically lose adenocarcinoma markers while acquiring strong, diffuse positivity for neuroendocrine markers (synaptophysin, chromogranin A, CD56). Whether small-cell transformation is simply associated with resistance or represents a direct causal mechanism remains uncertain. Nonetheless, these molecular and histological changes provide essential diagnostic evidence of transformation and highlight the importance of repeat biopsy and molecular profiling at progression. A concise summary of the key morphologic features and commonly used immunohistochemical markers distinguishing lung adenocarcinoma from transformed SCLC and squamous phenotypes is provided in Figure 1.

The clinical course of patients with SCLC transformation has been clarified by a multi-institutional retrospective analysis by Marcoux et al. (*n* = 67) [31]. In this cohort, 67% of patients harbored exon 19 deletions at diagnosis, and all had received at least one EGFR-TKI prior to transformation. The median time from diagnosis to transformation was 17.8 months, with a median survival after transformation of only 10.9 months [31]. Consistent with earlier reports, all transformed tumors retained their original *EGFR* mutations. Notably, 79% of patients (15 out of 19) who were T790M-positive before transformation lost T790M at the time of SCLC emergence, suggesting both a loss of EGFR dependency and potential resistance to subsequent EGFR-TKI therapy. Additional alterations in *TP53*, *RB1*, and *PIK3CA* were also frequently observed, reinforcing their role in driving lineage plasticity.

Complementing these findings, a multi-institutional Korean study by Lee et al. systematically characterized clonal history and genetic predictors of transformation [40]. The investigators screened 206 *EGFR*-mutant LUAD patients who underwent repeat biopsy at progression, along with additional cases collected from three other institutions. Clinically, transformation was observed across a broad demographic spectrum, most commonly in never-smokers with either exon 19 deletions or L858R mutations. Although the study did not report a pooled median time-to-transformation, the clinical timeline was broadly consistent with prior cohorts, as all patients developed transformation following TKI exposure. Importantly, concomitant inactivation of *RB1* and *TP53* in pre-treatment adenocarcinoma strongly predicted subsequent SCLC transformation (odds ratio 131, 95% CI 19.9–859). In contrast, demographic and clinical variables such as age, sex, smoking status, *EGFR* mutation subtype, and prior use of third-generation TKIs were not significantly associated with risk [40].

It is also important to recognize that the prevalence of histologic transformation is higher following resistance to third-generation EGFR-TKIs than to first- or second-generation inhibitors. Given the high selectivity of osimertinib for T790M, loss of T790M is a common observation at the time of osimertinib resistance [51]. Instead, alternative resistance mechanisms, including *MET* amplification and histologic transformation, are more frequently observed. In a study by Oxnard et al., 15% (6 of 41) of patients who developed resistance to second-line osimertinib exhibited SCLC transformation [52]. Similarly, in a cohort with paired pre- and post-treatment samples from patients with first-line osimertinib resistance, histologic transformation was reported in 15% (9 of 62) of cases, with squamous (*n* = 5) transformation being more common than SCLC (*n* = 3) [53].

Beyond differences in prevalence, emerging data suggest that histologic transformation following third-generation EGFR-TKIs may reflect distinct evolutionary trajectories compared with earlier-generation inhibitors. First- and second-generation EGFR-TKIs commonly select for on-target resistance through acquisition of T790M, preserving EGFR dependency and limiting the emergence of lineage plasticity-driven escape. In contrast, osimertinib exerts potent suppression of both activating *EGFR* mutations and T790M, creating a strong evolutionary bottleneck that favors EGFR-independent resistance mechanisms, including histologic transformation and bypass signaling.

Consistent with this model, loss of T790M at osimertinib resistance is frequently observed and is often accompanied by the emergence of alternative phenotypes, including SCLC and squamous transformation, suggesting broader lineage plasticity under third-generation selective pressure. These observations raise the possibility that osimertinib may preferentially select for pre-existing plastic subclones or accelerate epigenetic reprogramming toward EGFR-independent states. However, direct comparative longitudinal studies remain limited, and further work is needed to determine whether transformation following osimertinib differs in timing, molecular dependencies, or reversibility compared with earlier-generation EGFR-TKIs.

Together, these studies define the clinico-pathological hallmarks of SCLC transformation: retention of the initiating *EGFR* mutation, acquisition of neuroendocrine morphology and markers, frequent loss of T790M at transformation, and enrichment for co-alterations in *TP53* and *RB1*. These features not only provide diagnostic confirmation but also highlight the molecular vulnerabilities that enable lineage plasticity and therapeutic resistance.

## 5. Challenges in the Detection of Histological Transformation

Despite advances in identifying histological transformation, several challenges persist in clinical detection. A major limitation is the inherent intratumoral heterogeneity (ITH), which can lead to sampling bias, particularly in small biopsies where the transformed component may be missed. Transformation often arises in spatially or temporally distinct clones, further reducing the likelihood of detection with a single-site biopsy [28,42]. Although re-biopsy of growing tumors at the time of clinical progression has become increasingly important for providing accurate prognostic information and guiding therapy, it is not always feasible in practice due to patient condition, anatomical constraints, or procedural risks [29,30]. These barriers contribute to the underdiagnosis of transformation, which also remains partly dependent on pathologist interpretation.

Moreover, accumulating evidence suggests that molecular or transcriptomic reprogramming may precede overt histologic change. For instance, Chua et al. reported 27% (4 out of 15) *EGFR*-mutant T790M-tumors exhibited widespread loss of adenocarcinoma lineage markers such as *NAPSA*, *NKX2-1*, *SFTA2*, and *SFTA3*, and increased expression of squamous or neuroendocrine marker genes, despite retaining adenocarcinoma morphology [38]. These findings support the concept of a “subclinical” or pre-morphologic transformation phase, in which lineage plasticity and epigenetic reprogramming occur well before transformation becomes histologically apparent. In some cases, lineage plasticity may not result in overt histologic changes but can still drive treatment resistance (Figure 1).

Collectively, these observations underscore the need for molecular approaches capable of capturing early signs of lineage plasticity, thereby enabling therapeutic intervention before irreversible histologic transformation occurs. Recent studies have demonstrated the feasibility of plasma-based detection strategies using epigenomic and regulatory profiling of cell-free DNA (cfDNA). El Zarif et al. showed that cfDNA epigenomic features, including histone post-translational modifications, DNA methylation, and chromatin accessibility, can distinguish transformed SCLC from *EGFR*-mutant lung adenocarcinoma with high accuracy, reflecting widespread epigenomic reprogramming associated with transformation [45]. Complementing this, Hiatt et al. developed a targeted cfDNA sequencing framework focused on transcriptional regulatory regions, enabling molecular subtyping of SCLC and accurate discrimination of transformation events following targeted therapy [54].

Taken together, these approaches highlight the potential of cfDNA-based epigenomic and transcriptional profiling to detect transformation at a pre-morphologic stage, addressing key limitations of tissue-based diagnostics in the setting of tumor heterogeneity.

## 6. Limitations of Current Treatment for SCLC-Transformed NSCLC

Even when transformation is identified, treatment options remain limited and largely unsatisfactory based on currently available clinical data. Management typically follows standard SCLC regimens, most commonly platinum–etoposide chemotherapy. However, data from case reports, prospective biopsy cohorts, and retrospective series have shown that transformed SCLC responds less favorably than de novo SCLC, with lower response rates and shorter PFS [15,30,31]. These observations suggest that transformed SCLC is a biologically distinct entity rather than a clinical analogue of de novo disease, underscoring the need for novel therapeutic strategies.

Attempts to improve outcomes with immunotherapy have also been disappointing across reported clinical cases and small series [55,56]. Although immune checkpoint inhibitors (ICIs) are now part of standard first-line therapy for extensive-stage SCLC (ES-SCLC), their benefit in transformed SCLC appears limited [57]. Recent network meta-analyses and real-world studies in treatment-naïve ES-SCLC populations have demonstrated modest improvements in overall survival with immune checkpoint inhibitor-based combination regimens compared with chemotherapy alone [58,59]. However, these benefits have been observed exclusively in de novo ES-SCLC and cannot be extrapolated to transformed SCLC. In contrast, available clinical series in transformed SCLC have reported low response rates to immunotherapy, likely reflecting the low tumor mutational burden and immunologically “cold” phenotype of *EGFR*-mutant tumors, even after transformation [31].

Similarly, re-challenge with EGFR-TKIs is generally ineffective once transformation has occurred, as reported across small case series and cohort studies. Although transformed tumors usually retain the original *EGFR* mutation, they lose EGFR signaling dependence, as reflected by the frequent loss of T790M and downregulation of EGFR expression at transformation [31,39,42]. Consequently, poor responses are frequently observed with TKI re-exposure in both small case series and larger cohorts.

Antibody-drug conjugates (ADCs) have recently emerged as potential therapeutic candidates for transformed or refractory SCLC. Sacituzumab Govitecan (SG, IMMU-132), a Trop-2–directed ADC conjugated with the topoisomerase I inhibitor SN-38, has demonstrated manageable safety and modest efficacy in heavily pretreated metastatic SCLC, achieving an overall response rate (ORR) of 14% and median PFS and OS of 3.7 and 7.5 months, respectively [60]. More recently, the phase 2 TROPiCS-03 trial (NCT03964727) confirmed the activity of SG as a second-line therapy in ES-SCLC, showing clinically meaningful responses irrespective of platinum sensitivity [61]. Beyond TROP-2, novel ADCs targeting alternative surface antigens are also under investigation. The B7-H3-directed ADC YL201 showed encouraging anti-tumor efficacy across multiple solid tumors in a phase 1/1b trial, with notable responses in ES-SCLC (ORR 63.9%) and lung adenocarcinoma (ORR 28.6%), accompanied by a manageable hematologic-toxicity profile [62]. Together, these findings highlight next-generation ADCs as a promising class of therapies in heavily pretreated SCLC populations, although their efficacy in transformed SCLC remains to be defined, with the potential to expand treatment options beyond conventional chemotherapy for SCLC-transformed and EGFR-resistant NSCLC.

In parallel, several emerging therapeutic strategies are being explored in SCLC and may hold relevance for transformed disease. Lineage-associated surface targets such as DLL3 are frequently upregulated following small-cell transformation, supporting the rationale for DLL3-directed therapies such as bispecific T-cell engagers, e.g., tarlatamab [63]. However, emerging real-world data in *EGFR*-mutant transformed SCLC suggest that clinical responses to tarlatamab are substantially lower (ORR 7%) than those observed in de novo SCLC (ORR 40%), highlighting important biological differences between these entities beyond DLL3 expression alone [64]. Additional approaches under investigation include targeting anti-apoptotic dependencies, such as BCL2, which has been implicated as a context-dependent vulnerability in preclinical models of SCLC transformation, as discussed under Section 7.5 [39,43].

Epigenetic dysregulation has been implicated in the regulation of lineage plasticity in SCLC and may represent a potential therapeutic avenue in transformed disease. The histone methyltransferase “enhancer of zeste homolog 2” (EZH2) is frequently upregulated following RB pathway inactivation in de novo SCLC and has been shown to promote malignant progression through aberrant PRC2-mediated transcriptional repression [65]. Preclinical studies demonstrate that EZH2 inhibition suppresses tumor growth in SCLC patient-derived xenograft models [66]; however, evidence supporting its activity specifically in *EGFR*-mutant transformed SCLC remains limited.

In contrast, histone deacetylase (HDAC) inhibition has more directly been implicated in histologic transformation and neuroendocrine transdifferentiation. Dysregulation of HDAC family members has been observed during NSCLC transformation, and preclinical studies suggest that HDAC inhibition can partially reverse lineage reprogramming. Notably, Oh et al. demonstrated that the HDAC inhibitor fimepinostat restores EGFR expression and enhances sensitivity to EGFR-TKIs in neuroendocrine-transformed *EGFR*-mutant models, supporting epigenetic reprogramming as a potentially reversible process [42]. Together, these findings suggest that epigenetic modulators, particularly HDAC inhibitors, may represent a rational therapeutic strategy for targeting lineage plasticity in transformed SCLC, supported primarily by preclinical evidence to date.

Despite these emerging advances, durable control of transformed disease remains elusive, emphasizing that therapeutic progress must be coupled with a deeper biological understanding.

## 7. The Need for Better Understanding via Available Data

The poor therapeutic outcomes of transformed SCLC underscore that clinical progress must be matched by a deeper understanding of the biological mechanisms driving transformation. With the growing availability of patient-derived samples, cell lines, genomic datasets, and emerging single-cell studies, researchers are now beginning to systematically dissect the genetic, epigenetic, and transcriptional programs underlying lineage plasticity and resistance evolution in *EGFR*-mutant NSCLC. These complementary resources provide a foundation for identifying early molecular events, mapping clonal dynamics, and uncovering therapeutic vulnerabilities unique to the transformed phenotype.

### 7.1. Genomic

One of the most compelling insights into the biology of transformation has come from studies tracing the evolutionary lineage of transformed tumors. The repeated observation that SCLC-transformed tumors retain their original *EGFR* mutations supports the hypothesis that they arise from pre-existing LUAD cells rather than representing de novo SCLC. In a pivotal study, Lee et al. reconstructed the clonal evolution of advanced *EGFR*-mutant LUADs that underwent SCLC transformation and demonstrated a common clonal origin with branched evolutionary trajectories, a finding later supported by Zhang et al. through genomic analyses of transformed tumors [40,41]. Remarkably, clonal divergence toward transformation occurred as early as before the initiation of EGFR-TKI treatment, with transformed clones showing complete inactivation of *TP53* and *RB1*, confirmed by immunohistochemistry. In a predefined cohort of *EGFR*-mutant LUAD patients (*n* = 65), concurrent loss of *TP53* and *RB1* conferred a 43-fold increased risk of SCLC transformation, positioning these alterations as early indicators of lineage switching [40].

These findings were further supported by Offin et al., who showed that *EGFR*-mutant LUADs harboring concurrent *TP53* and *RB1* alterations were at significantly higher risk of transformation, exhibited poorer clinical outcomes, and displayed additional genomic features such as whole-genome doubling and APOBEC-mediated mutagenesis [46]. These events may represent early genomic determinants of lineage plasticity.

In addition to genomic retention of *EGFR* mutations, recent clinical analyses have shown that *EGFR*-mutant proteins and EGFR-related signaling pathways may remain detectable at the time of SCLC/NEC transformation and in subsequent re-biopsy samples, highlighting a potential disconnect between lineage identity and oncogenic pathway activity [41].

Collectively, these data highlight the critical role of early genetic alterations (concurrent loss of *TP53* and *RB1*) in predisposing tumors to neuroendocrine transformation and underscore the importance of integrating molecular profiling into risk assessment strategies.

### 7.2. Transcriptomic and Epigenetic

While genomic alterations, such as *TP53*, *RB1*, and *PIK3CA* mutations, are frequently observed, recent evidence suggests that SCLC transformation is primarily driven by transcriptional reprogramming rather than mutational events alone. Transcriptomic profiling of transformed tumors has demonstrated upregulation of genes associated with the PRC2 complex, *PI3K*/*AKT*, and NOTCH signaling pathways [28].

Epigenomic studies reinforce the role of widespread chromatin remodeling in lineage plasticity. Differential methylation profiling has shown hypomethylation of transcription factors (TF) binding motifs linked to neuroendocrine differentiation, Wnt signaling activation, stemness, and epithelial–mesenchymal transition, whereas hypermethylation was observed in motifs associated with MAPK signaling and Wnt pathway suppression [28]. These epigenetic changes alter TF motif accessibility, allowing tumor cells to dynamically reprogram lineage identity under therapeutic pressure. Additional changes in histone modifications, DNA methylation, and chromatin accessibility further contribute to the silencing of epithelial lineage genes and the adoption of a neuroendocrine phenotype [45].

Collectively, these findings highlight the limitations of strategies targeting genetic mutations alone and emphasize the need for therapies that address transcriptional and epigenetic plasticity in SCLC-transformed tumors.

Importantly, emerging single-cell and spatial studies suggest that these transcriptional and epigenetic changes do not occur abruptly but instead unfold through intermediate cellular states. These transitional populations often retain partial adenocarcinoma identity while acquiring neuroendocrine or basal-like features, consistent with a model in which early genomic alterations, such as concurrent *TP53* and *RB1* loss, create a permissive chromatin landscape that facilitates subsequent epigenetic and transcriptional reprogramming mediated by regulators such as PRC2 and NOTCH signaling. In this framework, epigenetic reprogramming acts downstream of genetic permissiveness to stabilize lineage switching rather than initiating it de novo.

### 7.3. Spatial Transcriptomic

Spatial transcriptomic profiling has further refined our understanding of intra-tumoral heterogeneity during histological transformation. In a recent study, Oh et al. performed spatially resolved transcriptome analysis on 59 tumor regions of interest from formalin-fixed, paraffin-embedded (FFPE) tissue sections obtained from 10 patients with *EGFR*-mutant NSCLC [42]. These samples encompassed diverse histologies, including adenocarcinoma, combined SCLC/NSCLC collected prior to EGFR-TKI treatment, and transformed SCLC (t-SCLC) after EGFR-TKI therapy. Remarkably, approximately 94% (15 out of 16) transformed components evolved into neuroendocrine-high subtypes (SCLC-A and SCLC-N), accompanied by a statistically significant decrease in EGFR expression (*p* < 0.001) at both transcriptomic and protein levels.

Importantly, the spatial organization of these transcriptional programs indicates that neuroendocrine-associated gene expression and epigenetic pathway activation can emerge in localized tumor regions prior to uniform histologic conversion, suggesting that molecular lineage reprogramming precedes and spatially anticipates overt morphologic transformation. The spatial coexistence of adenocarcinoma and neuroendocrine-high regions within the same tumor is consistent with a branched evolutionary architecture, in which genetically related subclones diverge transcriptionally to adopt distinct lineage states under therapeutic pressure [40]. Within this framework, spatially localized regions exhibiting partial neuroendocrine activation but incomplete histologic conversion may represent putative transitional niches that mark early stages of lineage switching.

Pathway analysis revealed epigenetic alterations in t-SCLC, and treatment with histone deacetylase (HDAC) inhibitors restored EGFR expression in both cell line and organoid models [42]. Furthermore, combining the HDAC inhibitor fimepinostat with osimertinib produced synergistic antitumor effects in vitro and in vivo. These findings point to a promising therapeutic strategy to reverse or suppress lineage transition by targeting epigenetic reprogramming.

The spatial coexistence of adenocarcinoma and neuroendocrine-high regions further supports a stepwise model of transformation, in which genetically primed clones undergo localized epigenetic and transcriptional reprogramming before achieving full neuroendocrine commitment.

### 7.4. Single Cell Transcriptomic

Single-cell transcriptomic studies provide an essential framework for connecting early genomic alterations with downstream transcriptional state changes by resolving intermediate cellular populations that are obscured in bulk analyses. For example, Zhang et al. performed integrated single-cell RNA sequencing across LUAD and LUSC tumors, identifying transcriptionally distinct alveolar type II (AT2) and basal-like subpopulations associated with disease progression and patient survival [67]. These data highlight the prognostic and biological relevance of cell type-specific transcriptional programs and reinforce the need for single-cell resolution when studying lineage plasticity.

In this context, Gardner et al. provided a lineage-based framework for understanding transformation [44]. Using genetically engineered mouse models combined with single-cell RNA sequencing, the authors demonstrated that lineage-specific oncogenic tolerance dictates whether histologic transformation can occur. AT2 cells, which commonly give rise to LUAD, tolerated EGFR-driven oncogenesis but were resistant to Myc-induced transformation. In contrast, pulmonary neuroendocrine cells were highly sensitive to Myc but intolerant to EGFR signaling. LUAD-to-SCLC transformation required concurrent loss of *RB1* and *TP53*, and in some contexts, loss of *PTEN* to allow Myc-driven transformation. The emergence of a basal-like, stem-like intermediate state facilitated this lineage switch. These findings underscore that transformation is not dictated solely by genetic or epigenetic alterations but is also constrained or permitted by intrinsic lineage compatibility.

While Gardner et al. provided valuable mechanistic insights, it is important to recognize that the cellular architecture and microenvironment differ significantly between mice and humans. As a result, the same genetic perturbations may yield different phenotypic outcomes across species. For example, the observed requirement for *PTEN* loss in AT2-driven transformation may reflect species-specific biology, given that human airways contain a greater diversity of epithelial subtypes not represented in mouse models.

Myc is a well-established reprogramming factor, and its overexpression has been shown to induce stem-cell-like states or reprogram mammary, prostate, and lung epithelial cells into neuroendocrine-like phenotypes. Whether transformed tumor cells remain dependent on MYC signaling for survival, however, remains unclear. This question warrants further study, particularly given the therapeutic implications of targeting MYC-driven plasticity.

Importantly, the single-cell approach employed by Gardner et al. was instrumental in uncovering the role of lineage context in shaping transformation outcomes. By resolving lineage relationships and transcriptional states at single-cell resolution, these studies provide a mechanistic framework in which histologic transformation proceeds through intermediate transcriptional states constrained by lineage context, rather than through an abrupt, binary phenotypic switch. However, direct single-cell analyses in human transformation remain limited, underscoring the need for studies that capture lineage plasticity in patient-derived samples.

### 7.5. Functional Studies

Functional studies have been instrumental in validating the mechanisms underlying histological transformation. In a landmark study, Niederst et al. generated patient-derived cell line models of *EGFR*-mutant LUAD that underwent SCLC transformation after EGFR-TKI treatment [39]. All transformed tumor and cell line samples exhibited loss of *RB1*, a phenomenon rarely observed in tumors that remained adenocarcinoma. Transformation was accompanied by a marked reduction in EGFR expression and increased expression of neuroendocrine markers such as ASCL1. Importantly, these transformed cells also displayed sensitivity to BCL-2 inhibition, a vulnerability characteristic of classical SCLC biology and one that has motivated interest in anti-apoptotic targeting strategies in transformed disease. These findings not only support the role of RB1 inactivation and EGFR downregulation in driving lineage plasticity but also illustrate that SCLC-transformed tumors acquire a distinct therapeutic profile no longer dependent on EGFR signaling.

In contrast, Ding et al. demonstrated that neuroendocrine differentiation induced by *SMAD4* and/or *RB1* inactivation in NSCLC models was not accompanied by increased expression of canonical SCLC therapeutic targets in cell line models, including BCL2 or DLL3, despite clear upregulation of neuroendocrine markers [43]. This finding suggests that acquisition of a neuroendocrine phenotype alone may be insufficient to confer classical SCLC drug dependencies.

Building on this, Quintanal-Villalonga et al. established a patient-derived xenograft (PDX) from an *EGFR*-mutant LUAD that subsequently transformed into SCLC [28]. The PDX maintained combined LUAD and SCLC histology through serial passaging, allowing in vivo therapeutic testing. In this model, AKT inhibition with samotolisib delayed tumor growth and synergized with osimertinib to suppress the LUAD component, although the neuroendocrine component persisted. This work demonstrates that transformed tumors contain co-existing lineages with distinct drug sensitivities and underscores the selective pressure of EGFR-targeted therapy in enriching neuroendocrine subclones.

In parallel, genetically engineered mouse models (GEMMs) have been used to replicate transformation under defined genetic perturbations. Gardner et al. showed that LUAD-to-SCLC transformation required concurrent loss of *RB1* and *TP53*, and in some contexts additional *PTEN* loss, to permit Myc-driven transformation [44]. These models also revealed the emergence of a stem-like intermediate state, highlighting how lineage compatibility constrains or enables plasticity.

Together, these functional systems, ranging from cell lines to organoids [42], PDXs, and GEMMs, offer indispensable tools for bridging molecular observations with therapeutic discovery. They demonstrate that transformed tumors are biologically distinct from both de novo SCLC and non-transformed *EGFR*-mutant LUAD and provide platforms for identifying novel therapeutic vulnerabilities.

### 7.6. Multi-Omics Study

Integrative multi-omics profiling has provided deeper insight into the biological underpinnings and therapeutic implications of lineage plasticity. In a comprehensive analysis of 59 *EGFR*-mutant NSCLCs resistant to first- or second-generation EGFR-TKIs but lacking the T790M mutation, Chua et al. performed whole-exome and transcriptome sequencing [38]. They observed pervasive transcriptional downregulation of adenocarcinoma lineage markers (*NAPSA*, *NKX2-1*, *SFTA2*, and *SFTA3*), validated orthogonally via multiplex immunohistochemistry. These tumors were enriched for *TP53* mutations, 3q amplifications, whole-genome doubling, and nonaging mutational signatures (smoking, APOBEC, and DNA repair). They also displayed an “immune-hot” phenotype with high PD-L1 expression and upregulation of PI3K/AKT signaling, suggesting potential vulnerabilities to PI3K inhibitors or immunomodulatory approaches.

Complementing this, Quintanal-Villalonga et al. integrated genomic, transcriptomic, epigenomic, and proteomic data from paired pre- and post-transformation samples as well as patient-derived xenografts [28]. Their analysis revealed that loss of chromosome 3p often preceded transformation and that PRC2, NOTCH, and PI3K/AKT pathways were consistently activated at both transcriptomic and proteomic levels. Importantly, pharmacologic inhibition of PI3K/AKT signaling in vivo delayed tumor growth and suppressed neuroendocrine transformation, underscoring these pathways as actionable mediators of plasticity.

A particularly valuable aspect of the Quintanal-Villalonga study was its use of mixed histology tumors, which enabled dissection of the molecular and phenotypic landscapes underlying coexistence and transition between LUAD and SCLC components. Such designs highlight the importance of studying transformation not as a binary switch but as a spectrum of intermediate states captured through multi-omics integration.

Beyond the canonical *TP53*/*RB1* axis, recent functional and genomic analyses have identified additional lineage-enabling alterations, such as *SMAD4* loss, which can promote neuroendocrine reprogramming through modulation of ASCL1-driven transcriptional programs, reinforcing the concept that transformation reflects convergent plasticity mechanisms rather than a mutation-specific phenomenon [43].

**Figure 2:**
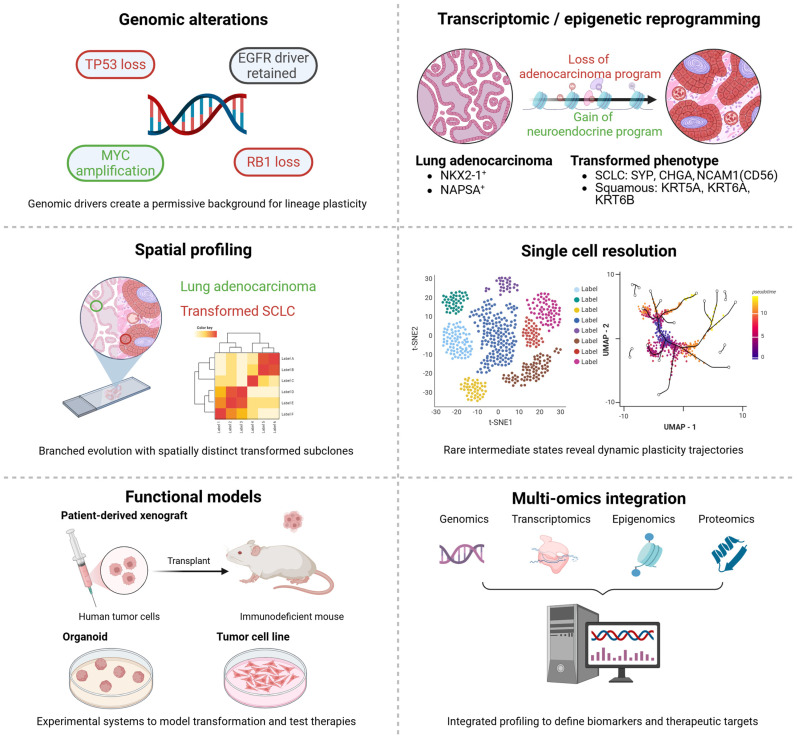
Approaches to studying histologic transformation in *EGFR*-mutant NSCLC. Genomic [40,46], transcriptomic/epigenetic [28,45], spatial [42], and single-cell profiling [44,67] reveal the molecular trajectories of lineage plasticity. Functional models [28,39,42,44] and multi-omics integration [28,38] validate mechanisms and uncover therapeutic vulnerabilities unique to transformed tumors. Created in BioRender. Skanderup, A. (2025) https://BioRender.com/yj5jz43 (accessed on 15 November 2025).

**Figure 3:**
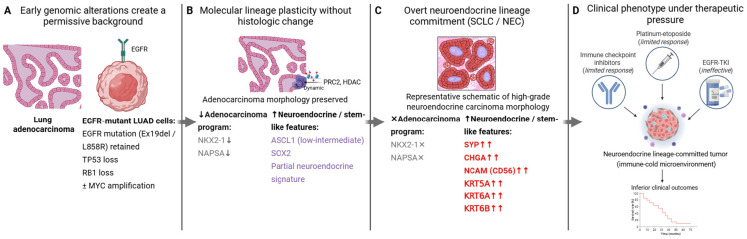
Integrated model of lineage plasticity-driven transformation in *EGFR*-mutant lung cancer. (**A**) Early genomic alterations, including *TP53*/*RB1* loss with retention of activating *EGFR* mutations, create a permissive background for lineage plasticity. (**B**) Molecular and epigenetic reprogramming can emerge without overt histologic change, characterized by partial loss of adenocarcinoma lineage programs and acquisition of stem-like or neuroendocrine features. (**C**) Progressive stabilization of these programs leads to overt lineage commitment and histologic transformation into high-grade neuroendocrine or alternative transformed phenotypes. (**D**) Lineage-committed tumors exhibit limited responsiveness to standard therapies and poor clinical outcomes under therapeutic pressure. Created in BioRender. Skanderup, A. (2025) https://BioRender.com/2caav28 (accessed on 15 November 2025).

Taken together, genomic, transcriptomic, epigenetic, spatial, and single-cell studies converge on a model in which histologic transformation is the product of both early enabling alterations, such as concurrent *TP53* and *RB1* loss, and dynamic reprogramming events that unfold under therapeutic pressure. A variety of experimental approaches, from bulk sequencing of patient tumors to spatial profiling and functional mouse models, have been applied to study transformation (Table 1). Spatial and single-cell analyses reveal that transformation often arises through branched evolution, with intermediate states bridging adenocarcinoma and neuroendocrine lineages. Functional models, including patient-derived cell lines, xenografts, and genetically engineered mouse models, have validated these mechanisms and provided platforms for testing therapeutic strategies. Finally, multi-omics integration highlights transformation as a continuum of lineage states rather than a binary switch, offering molecular entry points for intervention before full histological change occurs. Together, these insights emphasize that transformation is not merely a rare complication of targeted therapy but a predictable manifestation of lineage plasticity, one that must be addressed through integrated detection strategies and tailored therapeutic approaches informed by emerging experimental and multi-omics frameworks (Figure 2) and summarized in an integrated transformation model (Figure 3).

## 8. SCLC Transformation Beyond EGFR-Mutant Disease

It remains unclear whether SCLC transformation represents a direct resistance mechanism or an independent evolutionary pathway. While most cases occur in *EGFR*-mutant LUADs treated with EGFR-TKIs, isolated reports have described transformation in EGFR-wildtype tumors following treatment with ALK inhibitors or PD1/PD-L1 immunotherapy [68,69,70]. In a recent study, Ding et al. confirmed that SCLC transformation can also occur in *EGFR*-wildtype NSCLCs, with an incidence rate of 9.73% [43]. In this cohort, *SMAD4*, *RICTOR*, and *RET* alterations were uniquely enriched in transformed tumors. Of particular note, *SMAD4* deficiency accelerated transformation in *EGFR*-wildtype, *TP53*-deficient NSCLC cells, suggesting that alternative molecular routes can drive neuroendocrine differentiation in the absence of *EGFR* mutation.

Histologic transformation has also been reported in other oncogene-driven NSCLCs. Small-cell transformation occurred in *ROS1* fusion-positive and *ALK*-rearranged lung cancers following TKI therapy. In a study of *ROS1* fusion-positive NSCLC resistant to ROS1 inhibitors, transformation to SCLC was observed with concurrent *RB1* and *TP53* inactivation and loss of *ROS1* fusion expression, suggesting convergent mechanisms of lineage plasticity and drug resistance [71]. Similarly, *ALK*-rearranged (most commonly *EML4*-*ALK*) adenocarcinomas have been shown, albeit primarily in case reports and small series, to undergo transformation to SCLC or large-cell neuroendocrine carcinoma (LCNEC) after ALK-TKI treatment [68,70,72,73,74,75,76,77,78,79]. Although the reported frequency of transformation in these subsets appears relatively low, approximately 2% (1 of 65) in *ROS1* fusion-positive and 0.8% (2/263) in ALK-TKI resistant cases [71], these findings underscore that histologic transformation is not restricted to *EGFR*-mutant disease but rather reflects a broader adaptive strategy enabled by lineage plasticity across diverse oncogenic contexts.

## 9. Conclusions

Histologic transformation in *EGFR*-mutant NSCLC exemplifies how lineage plasticity can undermine targeted therapy by enabling tumors to escape oncogene dependence through the adoption of alternative histology. Although first recognized as a rare clinical phenomenon, accumulating evidence has established SCLC transformation as a reproducible and clinically significant mechanism of resistance, characterized by retention of the initiating *EGFR* mutation, acquisition of neuroendocrine features, and aggressive clinical behavior.

Despite its clinical importance, transformation remains underdiagnosed due to intratumoral heterogeneity, biopsy limitations, and the static nature of FFPE sampling. Standard therapies yield only transient benefit, underscoring the urgent need for strategies informed by the unique biology of transformed tumors. Emerging genomic, transcriptomic, epigenetic, spatial, and single-cell studies have begun to reveal the molecular trajectories that foreshadow transformation and highlight potential therapeutic vulnerabilities. Functional models and multi-omics integration are now providing frameworks to test interventions that may intercept or reverse lineage switching.

Several limitations should be considered when interpreting the findings summarized in this review. Much of the available evidence on histologic transformation in NSCLC is derived from retrospective cohorts, small case series, and post-progression biopsies, which may underestimate the true prevalence of transformation and limit causal inference. In addition, because lineage switching represents a continuum rather than a binary event, intermediate molecular states may be missed by routine histologic assessment, particularly in the setting of limited tissue availability or single-time-point sampling. Although single-cell, spatial, and functional studies have provided critical mechanistic insights, many of these data originate from preclinical models, and longitudinal analyses in human tumors remain limited. Variability in diagnostic criteria and analytic approaches across studies further complicates comparisons of reported transformation frequencies and molecular signatures. Finally, therapeutic evidence specific to transformed SCLC remains sparse, with most treatment paradigms extrapolated from de novo disease, underscoring the need for prospective studies focused on lineage-transformed tumors.

Importantly, transformation is not restricted to *EGFR*-mutant disease but represents a broader adaptive strategy observed across oncogenic contexts, reinforcing lineage plasticity as a central theme in resistance biology. Moving forward, integrating molecular profiling into clinical workflows, developing biomarkers of early plasticity, and designing therapies tailored to lineage-switched states will be critical to improving outcomes.

## Figures and Tables

**Table 1:** Overview of clinical and experimental studies on histologic transformation in NSCLC. Summary of major cohorts and experimental models, including sample type, species, and reported prevalence. All studies are based on primary data from patient cohorts or preclinical models. Prevalence values represent the number of *EGFR*-mutant (unless specified) transformed cases among the total analyzed (n/N, %). For preclinical mouse studies, sample size is not applicable.

Study	Species	Sample Type	Molecular Data Type *	Paired Samples	Sample Size/ Prevalence
Sequist et al. (2011) [14]	Human	FFPE tumor tissue	DNA	Yes	5/37 (14%)
Yu et al. (2013) [15]	Human	Fresh-frozen or FFPE tumor tissue	DNA, Protein	Yes	4/155 (3%)
Niederst et al. (2015) [39]	Human/Mouse	OCT-embedded frozen tumor tissue	DNA, RNA, Protein	Yes	11
Lee et al. (2017) [40]	Human	Fresh-frozen or FFPE tumor tissue	DNA, Protein	Yes	21
Quintanal-Villalonga et al. (2021) [28]	Human	Fresh-frozen tumor tissue	DNA, RNA, Protein	Yes	3
Chua et al. (2021) [38]	Human	Fresh-frozen tumor tissue	DNA, RNA	No	1/59 (1.7%)
Marcoux et al. (2021) [31]	Human	Tumor tissue (unspecified)	DNA	No	58
Zhang et al. (2023) [41]	Human	FFPE tumor tissue	DNA, Protein	Yes	7 (DNA), 11 (Protein)
Oh et al. (2024) [42]	Human	FFPE tumor tissue	RNA, Protein	Yes	10
Ding et al. (2024) [43]	Human	FFPE tumor tissue	DNA, RNA, Protein	Yes	EGFR wildtype: 11/113 (9.7%) EGFR-mutant: 13/230 (5.7%)
Gardner et al. (2024) [44]	Mouse	FFPE mouse lung tissue	DNA, RNA, Protein	No	N/A (mouse model)
El Zarif et al. (2024) [45]	Human/Mouse	Patient plasma, fresh-frozen tumor tissue, and PDX samples	DNA, RNA	No	4
Offin et al. (2019) [46]	Human	Tumor tissue (unspecified)	DNA	No	7/39 (18%)
Fujimoto et al. (2022) [37]	Human	Tumor tissue (unspecified)	DNA	No	74/2624 (2.8%)

* Molecular data type refers to the omics layer analyzed in each study; “DNA” includes genomic or methylation profiling; “RNA” includes gene expression or transcriptomic analyses; “Protein” includes proteomic, immunohistochemical, or signaling analyses.

## Data Availability

No new data were created or analyzed in this study. Data sharing is not applicable to this article.
